# INTERGENERATIONAL COURSE ABROAD EXPLORES AGE INCLUSIVITY IN SCOTLAND

**DOI:** 10.1093/geroni/igad104.0085

**Published:** 2023-12-21

**Authors:** Andrea June, Carrie Andreoletti

**Affiliations:** Central Connecticut State University, New Britain, Connecticut, United States; Central Connecticut State University, New Britain, Connecticut, United States

## Abstract

In 2017, Central Connecticut State University (CCSU) joined the Age-Friendly University (AFU) global network. Guided by the AFU framework to promote age inclusivity in higher education, many programs and services at CCSU have been expanded to encourage the participation of older adults in all core activities of the university. The authors have a particular interest in fostering intergenerational learning opportunities, for which there are reciprocal benefits (e.g., Andreoletti & Howard, 2016). With the goals of promoting intergenerational connection and global age-inclusivity, we developed a course abroad to Scotland called Global Aging & Age-Friendly Initiatives. Prior to traveling, students are introduced to theory and research in lifespan development and to the World Health Organization’s (WHO) definition of healthy aging and domains of livability aimed at enhancing well-being and longevity. Livability community efforts in the USA are explored so that similarities and differences can be examined while in Scotland. During 9 days of travel, students are exposed to urban and rural areas of Scotland, noting the accessibility of public transportation, the availability of green spaces, the cultural attitude toward older adults, and other domains of livability. They will also hear from local academics and community organizers invested in promoting engagement across the lifespan. In June 2023, eight age-diverse learners will travel with the authors. In addition to providing more detail on the development and implementation of the course abroad, this presentation will share feedback from students and international collaborators with the goal of encouraging other AFUs to develop similar opportunities.

